# Designing a use-error robust machine learning model for quantitative analysis of diffuse reflectance spectra

**DOI:** 10.1117/1.JBO.29.1.015001

**Published:** 2024-01-11

**Authors:** Allison Scarbrough, Keke Chen, Bing Yu

**Affiliations:** a Marquette University and Medical College of Wisconsin, Joint Biomedical Engineering Department, Milwaukee, Wisconsin, United States; b Marquette University, Computer Science Department, Milwaukee, Wisconsin, United States

**Keywords:** diffuse reflectance spectroscopy, optical properties, machine learning, cancer detection

## Abstract

**Significance:**

Machine learning (ML)-enabled diffuse reflectance spectroscopy (DRS) is increasingly used as an alternative to the computation-intensive inverse Monte Carlo (MCI) simulation to predict tissue’s optical properties, including the absorption coefficient, $\mu_{a}$ and reduced scattering coefficient, $\mu_{s}^{\prime }$.

**Aim:**

We aim to develop a use-error-robust ML algorithm for optical property prediction from DRS spectra.

**Approach:**

We developed a wavelength-independent regressor (WIR) to predict optical properties from DRS data. For validation, we generated 1520 simulated DRS spectra with the forward Monte Carlo model, where $\mu_{a}=0.44$ to $2.45\;{\mathrm{cm}}^{-1}$, and $\mu_{s}^{\prime }=6.53$ to $9.58\;{\mathrm{cm}}^{-1}$. We introduced common use-errors, such as wavelength miscalibrations and intensity fluctuations. Finally, we collected 882 experimental DRS images from 170 tissue-mimicking phantoms and compared performances of the WIR model, a dense neural network, and the MCI model.

**Results:**

When compounding all use-errors on simulated data, the WIR model best balanced accuracy and speed, yielding errors of 1.75% for $\mu_{a}$ and 1.53% for $\mu_{s}^{\prime }$, compared to the MCI’s 50.9% for $\mu_{a}$ and 24.6% for $\mu_{s}^{\prime }$. Regarding experimental data, WIR model had mean errors of 13.2% and 6.1% for $\mu_{a}$ and $\mu_{s}^{\prime }$, respectively. The errors for MCI were about eight times higher.

**Conclusions:**

The WIR model presents reliable use-error-robust optical property predictions from DRS data.

## 1 Introduction

### 1.1 Diffuse Reflectance Spectroscopy for Cancer Detection

There is an unmitigated disparity in timely detection of cancer in high-resource settings versus low-resource settings. Patients that live in low-resource settings, such as inner cities, rural areas, or lower-to-middle-income countries, are at a higher risk of facing late-stage cancer diagnoses and higher mortality rates from this family of diseases.^1^^–^^3^ Cervical cancer especially highlights this disparity, as ∼90% of cases occur in low-resource settings.^4^ This is, in part, caused by the fact that significant patient-to-clinician follow-up is required to obtain a diagnosis. Further, standard procedures for diagnosing cancer require rigorous sufficient healthcare resources and infrastructure. Timely access to clinicians and laboratory resources are often not feasible for those living at or below the poverty line.^1^^,^^2^ Thus, there is a need to develop a means of cost-efficiently detecting cancer at the point-of-care, giving special consideration to the logistical challenges in low-resource clinical settings. To accomplish this, many groups have looked to visible diffuse reflectance spectroscopy (DRS) as a means of capturing “optical biopsies” of suspicious lesions. There are many advantages to this “optical biopsy” approach. For example, this imaging can be done in an outpatient point-of-care setting, and equipment to obtain these biopsies, such as a visible optical spectrometer, is relatively inexpensive. For instance, the DRS system used to collect the data presented here costs <$2500 USD^5^ and is sensitive to many changes in the tissue microenvironment that occur during malignancy. One example of these changes includes increased angiogenesis, which manifests as abnormally high absorption coefficient, $\mu_{a}$. Another example is breakdown of the extracellular matrix within the tumor microenvironment, which causes an abnormally low reduced extinction coefficient, $\mu_{s}^{\prime }$.^6^

The specific methods for acquiring optical spectroscopy data vary. Generally, the process involves exposing biological tissue (either *in vivo* or *ex vivo*) to specific wavelengths of light. Then, a spectrometer is used to measure the optical signal that is either transmitted through or reflected from the tissue. Data analysis can be done using a numerical approach, such as an inverse Monte Carlo (MCI) model, which is widely considered the “gold standard” of modeling photon transport in turbid media.^7^^–^^9^ Other approaches include passing data through a lookup table,^10^ or through a machine learning model^11^^–^^24^ to predict tissue optical properties. Recently, sophisticated neural networks (NNs) and traditional machine learning methods have been favored for DRS analysis. This is because numerical approaches, such as the MCI model, are computationally intensive. Machine learning methods, on the other hand, can be faster and less computationally demanding than these numerical techniques.

### 1.2 Artificial Intelligence for DRS Analysis

The increased speed provided by AI-enabled spectroscopy is compelling in a clinical setting, due to its ability to provide results at the point-of-care, thus minimizing patient-to-clinician follow-up. This is done using either an NN approach,^11^^–^^14^^,^^17^^–^^24^ and/or a traditional machine learning approach^15^^,^^16^^,^^20^^,^^21^ (Table 1). When NNs are used, the raw, or minimally pre-processed, spectrum is the model’s input. The model autonomously determines features to use to predict optical properties ($\mu_{a}$ and $\mu_{s}^{\prime }$) from the input spectrum. In the traditional machine learning approach, features, such as the spectral intensity at a given wavelength, are manually selected by the programmer for model input. Then the algorithm uses those manually selected features to solve this predictive modeling problem.

**Table 1:** Previous work towards machine-learning-enabled spectroscopy for optical property prediction. Groups with a “Y” under “added noise?” introduced Gaussian or similar noise to data. Abbreviations: multilayer perceptron (MLP), absorption coefficient ($\mu_{a}$), reduced extinction coefficient ($\mu_{s}^{\prime }$), hemoglobin concentration ([Hb]), spatially resolved diffuse reflectance spectra (SRDRS), and random forest regression (RFR). Gradient boosting regressor (XGboost).

First author	Training data type	Training size	Testing size	Added noise?	Models	Prediction errors
Yudovsky^11^	Simulated DRS data	50,000	10,000	Y	Four-layer MLP	$\mu_{a}$: 8.0% to 14.7%
						$\mu_{s}^{\prime }$: 3.8% to 4.3%
Hokr^12^	Simulated DRS data	295,598	10,000	N	Five-layer MLP	$\mu_{a}$: 15%
						$\mu_{s}^{\prime }$: 30%
Fredriksson^13^	Simulated DRS data	100,000	100,000	Y	Three-layer MLP	[Hb]: 5.1%
						Oxygen sat: 13%
Kienle^14^	Simulated DRS data	120	13	N	Three-layer MLP	$\mu_{a}$: 14%
						Transport scattering: 2.6%
Panigrahi^15^	Simulated DRS data	$10^{6}$	$10^{6}$	N	RFR	$\mu_{a}$: 0.56%
						$\mu_{s}^{\prime }$: 0.13%
Wirkert^16^	Simulated multispectral images	15,000	5000	Y	RFR	$V_{\mathrm{Hb}}$: 5.4% to 5.5%
Chen^17^	Simulated DRS data	6100	6100	N	Three-layer MLP	$\mu_{a}$: 0.3%
						$\mu_{s}^{\prime }$: 0.6%
Jager^18^	Simulated SRDRS data	2000	2000	Y	Three-layer MLP	$\mu_{a}$: 6.1%
						$\mu_{s}^{\prime }$: 2.9%
Jager^19^	Simulated SRDRS curves	100	410	Y	Three-layer MLP	$\mu_{a}$: 4.4% to 8.7%
						$\mu_{s}^{\prime }$: 2.1% to 3.3%
Manojlovic^20^	Simulated hyperspectral images	80,000	20,000	N	Three-layer MLP, CNN, and RFR	Predicted spectra versus actual spectra disagreement: 0.003% to 0.009%
Nguyen^21^	Simulated DRS data	10,000	30,000	Y	Four-layer MLP, RFR, and XGboost	$\mu_{s}^{\prime }$: 6.88%
Zhang^22^	Simulated + phantom SRDRS data	32 simulations + 23 phantoms	10 simulations + 11 phantoms	N	Three-layer MLP	$\mu_{a}$: 6.0% to 9.0%
						$\mu_{s}^{\prime }$: 3.0% to 4.5%
Tsui^23^	Simulated SRDRS data	21,000	9000	Y	Four-layer MLP	$\mu_{a}$: 2.2% to 3.4%
						$\mu_{s}^{\prime }$: 2.6% to 25.9%
Farrell^24^	Simulated SRDRS data	800	100	Y	Three-layer MLP	$\mu_{a}$: <7.0%
						$\mu_{s}^{\prime }$: <7.0%

The main advantage of employing an NN for optical property prediction is that the feature extraction is done automatically. The expense of this is the requirement for more data to train the model. Additionally, the trained model itself is more computationally demanding. This is problematic in biomedical applications, as accessibility to a large enough training dataset to accurately train such a model without overfitting is scarce. An additional expense to the NN approach is its “black box” nature. Since the features are automatically selected, the features used to analyze the dataset may or may not be relevant to the posed scientific problem/question. This phenomenon is described by Tulio-Ribeiro et al.,^25^ where a deep learning model automatically selected features from images of wolves and Alaskan Husky dogs for classification. This model made classification decisions based on the image background (i.e., presence/absence of snow), rather than features relevant to the classification task. This artifact of models that automate feature extraction poses challenges to their translatability to high-stakes setting, such as in medical applications.

As a result, models that use manually selected features to predict a target variable present a compelling alternative in certain specific applications. Benefits of these models, over NNs, include that they tend to require less training data to reliably predict their target variables, and are more computationally lightweight. This comes at the cost of lessened ability to perform complicated classification and regression tasks.

### 1.3 Current Clinical Translatability of DRS and AI-enabled DRS

While the theoretical benefits of DRS and AI-enabled DRS are compelling, its clinical translatability, especially in low-resource settings, is limited. Previous studies heavily rely on training their models with simulated data that is either perfect, or noisy, but otherwise perfect.^11^^–^^24^ The justification for adding noise to these datasets is to simulate the impact of device noise on data acquisition. These studies fail to acknowledge that device noise is not the only common artifact present in experimental and clinical datasets. For example, the U.S. Food and Drug Administration estimates that as many as one in three device failures leading to adverse medical outcomes are not caused by an issue with the device itself.^26^ Conversely, they were caused by improper use of a medical device (defined as “use-error”).^26^ These errors are not necessarily caused by a lack of proper training. For example, a 2003 study found that of 1000 hand surgeons—expert practitioners with years of specialized medical training—“20% of them admitted to having operated on the wrong site at least once in their career.”^26^ Especially in a low-resource clinical setting, funding and resources to properly train clinicians on the proper use of every medical device they use may not be guaranteed. Beyond this, in both high and low-resource clinical settings, clinicians are often forced to make compromises regarding the use of various medications and medical devices. Specific to AI-enabled DRS analysis, it is important to anticipate the artifacts that common use-errors may introduce to DRS spectra and incorporate these anticipated artifacts in the training process.

Three major types of spectral artifacts that are commonly caused by use-errors are: Gaussian noise, spectral intensity fluctuations, and wavelength miscalibrations. Gaussian noise can be caused by noise in the camera, the light source, or the room light. Noise is not technically caused by use-error but is included in this discussion because it is a universal artifact of experimental data and often manifests as Gaussian-type noise. Because of its commonality in all experimental datasets, some groups have incorporated noise into the training and testing process of their algorithms (Table 1).^11^^,^^13^^,^^16^^,^^18^^,^^19^^,^^21^^,^^23^^,^^24^

Intensity fluctuations can be caused by improper probe-to-tissue contact, a light source not being properly warmed up prior to use, or similar use-errors. These artifacts can manifest as a spectrum’s intensity being falsely scaled up or down, either systematically (i.e., by the same amount throughout the entire spectrum), or in a wavelength-dependent manner. This artifact can be caused by failure to properly warm or charge up a light source or its battery before use. This error is common among those who do not have specialized training in optics, such as clinicians.

Wavelength miscalibrations can be caused by rough handling of the system, accidentally dropping/nudging the system, infrequent wavelength calibration, and similar use-errors. It is usually recommended that this use-error be avoided by regularly calibrating a spectrometer with a calibration lamp (i.e., a neon lamp). However, because this process is time consuming and expensive, some clinics, especially resource-limited clinics, may deviate from the required routine calibration schedule.

No other groups, to our knowledge, have investigated these three types of use-errors, and the impact their incorporation/absence in a training dataset has on the accuracy of popular predictive models for optical property prediction. It is problematic that these use-errors are not regularly incorporated into the training and testing processes of machine learning algorithms because datasets that are void of these errors are not representative of “real” experimental/clinical data. As a result, any resulting models, which are well documented to be extremely sensitive to bias in the training dataset,^27^ will likely produce inaccurate results if the data does not conform to the biased trends of the training dataset. Some groups partially mitigate the need for empirical wavelength-by-wavelength calibration through leveraging the shape and slope of spectra to arrive at optical property predictions.^14^^,^^18^^,^^23^ The vast majority of these groups apply this framework to spatially resolved diffuse reflectance spectra (SRDRS).^14^^,^^18^^,^^23^ This generally involves optimizing detectors to capture spectra at one or two specific wavelengths, rather than across a broad range, with a fiber optic probe.^14^ For instance, Kienle et al. calibrated SRDRS data at 633 and 751 nm^18^ and Jager et al. used 662 nm instead.^23^ Groups that use SRDRS to train machine learning models for optical property detection use the intensity and shape of SRDRS signal at a given wavelength as input into the algorithm. This approach incompletely solves the wavelength dependency problem because the system must be regularly calibrated to ensure that the signal detected by the SRDRS device is truly collecting data from the wavelength(s) of interest.

In addition, previously reported models are either completely trained, tested, and validated on simulated data, or use a minimal amount of experimental data (<40 datapoints, all collected in one session) for algorithm training/validation.^11^^–^^24^ This is problematic because experimental data is not likely to be perfectly aligned with theoretical data,^14^ and the reason is not always obvious. This is especially true when collecting data from biological tissue that is largely variable subject-by-subject and even day-by-day within one subject. Because of this, it is important to incorporate a significant body of experimental data into training for a given model.

There is an unmet need for a spectral analysis algorithm that anticipates common use-errors that are involved with humans and/or non-optical experts using optical equipment for data collection. Further, there is an unmet need to incorporate a large body of experimental data for training and validation to make models more adaptive to a realistic clinical environment. The research presented here addresses this issue by developing a lightweight machine learning method of predicting optical properties from DRS data. This is accomplished using wavelength-independent features in a model called the wavelength-independent regressor (WIR model). This novel analysis method has been rigorously trained and validated using both simulated spectra and phantom data collected on a previously described portable DRS system.^5^ This technique is novel in that: (1) it designs and extracts a combination of wavelength-independent features that have not been used by other studies; (2) it is rigorously trained and tested on a simulated dataset that incorporates spectral artifacts due to realistic use-errors; and (3) it is further rigorously trained and tested using 882 experimental DRS spectra collected from 170 tissue-mimicking hemoglobin (Hb) phantoms—the largest experimental dataset, to our knowledge, to be incorporated into DRS-based machine learning techniques thus far.

## 2 Methods

### 2.1 Smart Microendoscope

**Fig. 1:**
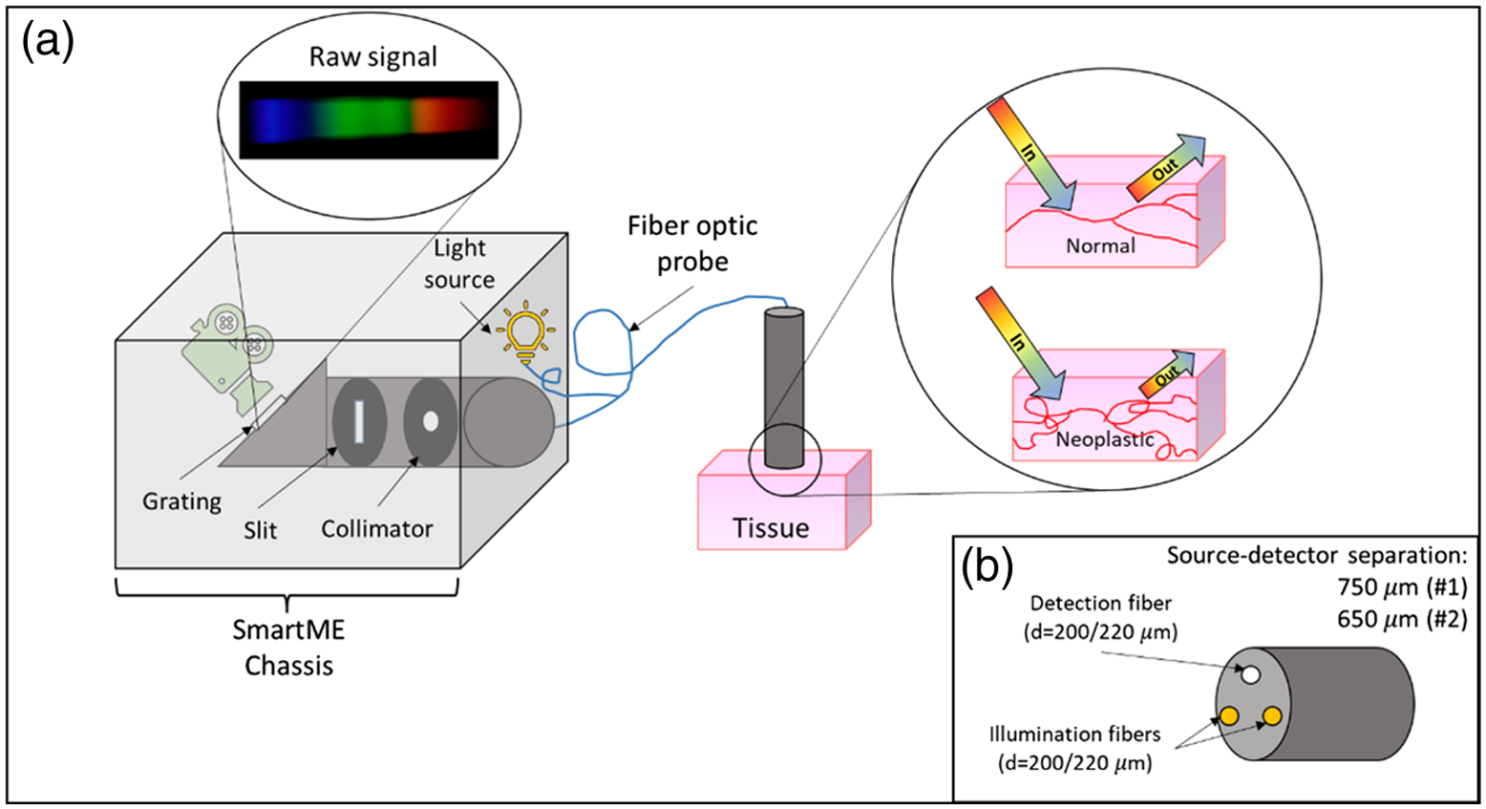
(a) DRS channel of the SmartME imaging system employed for data collection. This DRS channel worked by exposing the tissue to visible light via fiber optic probe and measuring the proportion of the illumination light this is reflected towards the probe. (b) Fiber optic probe geometry at the distal end.

The algorithm presented here analyzes DRS data collected using different versions of a previously described smart microendoscope (SmartME) imaging system for use in low-resource settings (Fig. 1).^5^ Briefly, the DRS channel of this system contained a visible white light source (Thorlabs MCWHF2; λ=450 to 630 nm), a homemade fiber optic probe for directing the optical signal to and from the tissue, and collimation/grating optics to produce a diffuse reflectance spectrum for imaging by a Samsung S7 camera. The resulting raw “spectrum” was an 8-bit RGB image that resembles that seen in Fig. 1(a). One of two probes was used to collect data for this study. Probe No. 1 had a source–detector separation (SDS) of 750  μm and Probe #2 had an SDS of 650  μm. All fiber diameters were 200/220  μm [Fig. 1(b)].

### 2.2 Generation and Analysis of Simulated Data

**Fig. 2:**
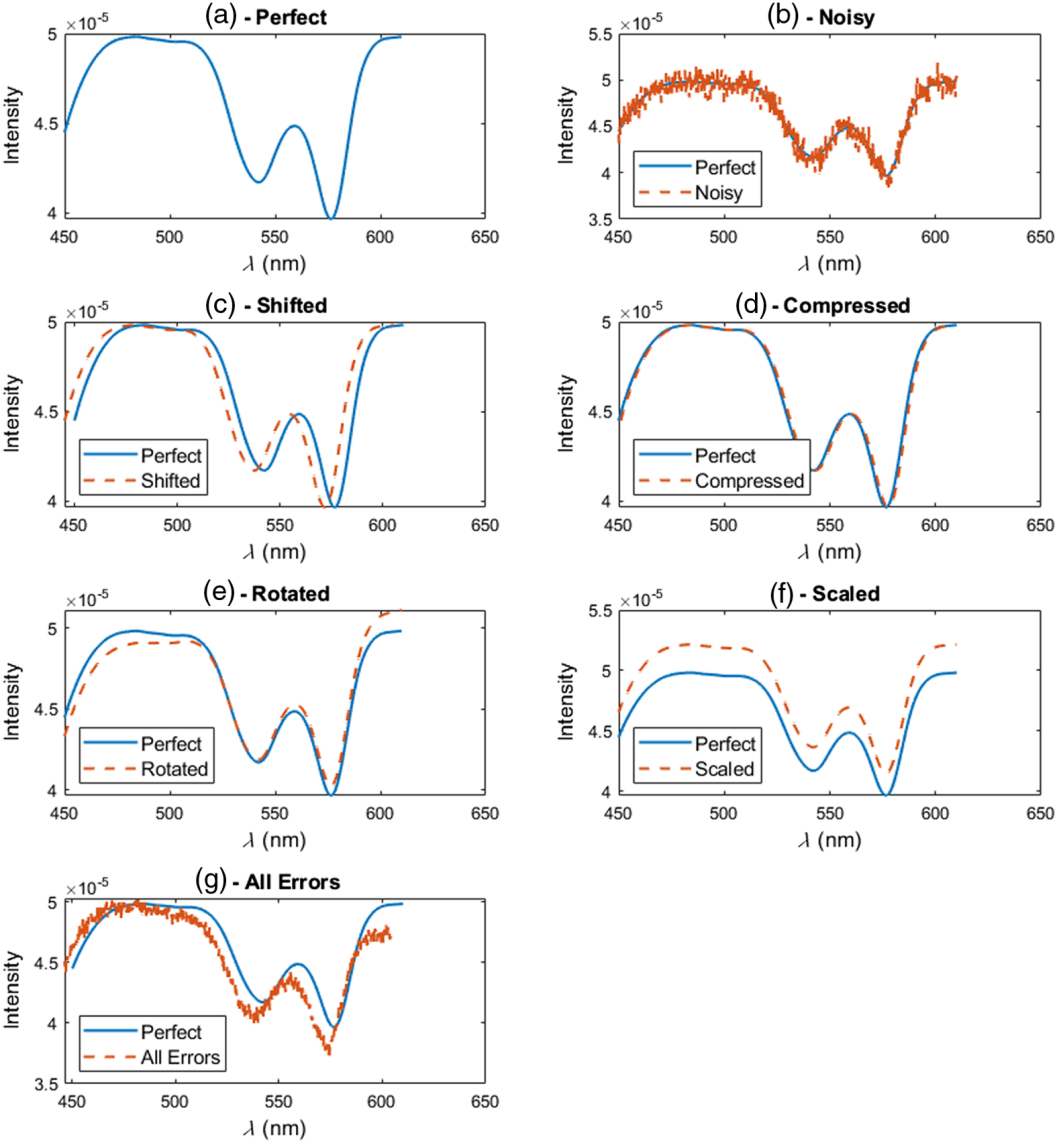
Example simulated spectra entered into the MCI, WIR, and MLP models for analysis. This includes data that are: (a) “Perfect” data, defined as the direct output of the forward Monte Carlo model, (b) “noisy,” but otherwise perfect, (c) wavelength miscalibrated, and (d) compressed, to simulate sensor misalignment. Other use-errors include spectra that have been (e) rotated, simulating another sensor misalignment error, (f) scaled up to simulate improper LED warm up period, or fiber bending induced fluctuation, and (f) data with all of the previously mentioned errors compounded on top of each other.

Simulations were generated using the forward Monte Carlo model described by Palmer.^28^ This was done to provide a “blank slate” for evaluating the WIR model’s performance. One can clearly see how the WIR model performs on each type of use-error in isolation, as the base dataset contained no experimental errors, other than those manually introduced into it. This also allowed one to clearly see which features are most affected (or conversely, most robust) to a given use-error. This also clearly demonstrated whether any given algorithm can learn and correct these use-errors. Seven total simulated datasets were generated, one dataset that was “perfect” [Fig. 2(a)], five datasets where one type of error was manually introduced, and one dataset on which all use-errors were compounded.

Error No. 1 included adding Gaussian noise using MATLAB’s awgn() function^29^ to the signal, such that the signal-to-noise ratio (SNR) was 35:1 [Fig. 2(b)] or 45:1 in the case of compounding all errors (Fig. 2(g)] due to issues with MCI convergence when all errors were compounded using the lower SNR. This SNR was decided quasi-arbitrarily, as it appeared qualitatively similar to the noise seen in other spectral data collected by the SmartME system.

Error No. 2 was a wavelength miscalibration in which spectra were compressed by 1 nm at each tail [Fig. 2(d)]. Error No. 3 was another wavelength miscalibration by shifting the spectra by 4.8 nm to the left [Fig. 2(c)]. This was determined using the SmartME device to capture a DRS spectrum from a neon lamp, at steady-state, at three different time points. The second measurement was taken 45 days after the first measurement, and a third measurement was taken 50 days after the first measurement. It was found that the neon peaks corresponding to the same wavelength, but taken on a different day, were off by as much as 12 pixels or 3.7 nm. An additional safety factor of 1.1 nm was added to our analysis, such that our data simulations were miscalibrated by as much as ±4.8  nm [Fig. 2(c)].

Error No. 4 was a wavelength-dependent intensity fluctuation, where the intensity of the spectrum was shifted up or down by a random amount, as much as 5%, as a function of spectral wavelength at each tail [i.e., the left tail was scaled up by 5%, the right tail scaled down by 5%, Fig. 2(e)]. This simulated the fluctuations in light source intensity that come with improper thermal management. Error No. 5 also simulated improper light source thermal management, leading to a systematic fluctuation in the intensity by as much as 5% [Fig. 2(f)]. Finally, Error No. 6 included compounding all errors on top of each other [Fig. 2(g)]. Each simulated dataset consisted of 1520 spectra, with $\mu_{a}$ ranging from 0.44 to $2.45\;{\mathrm{cm}}^{-1}$ (average step size: $0.007\;{\mathrm{cm}}^{-1}$), and $\mu_{s}^{\prime }$ ranging from 6.53 to $9.58\;{\mathrm{cm}}^{-1}$ (average step size: $0.008\;{\mathrm{cm}}^{-1}$).

**Fig. 3:**
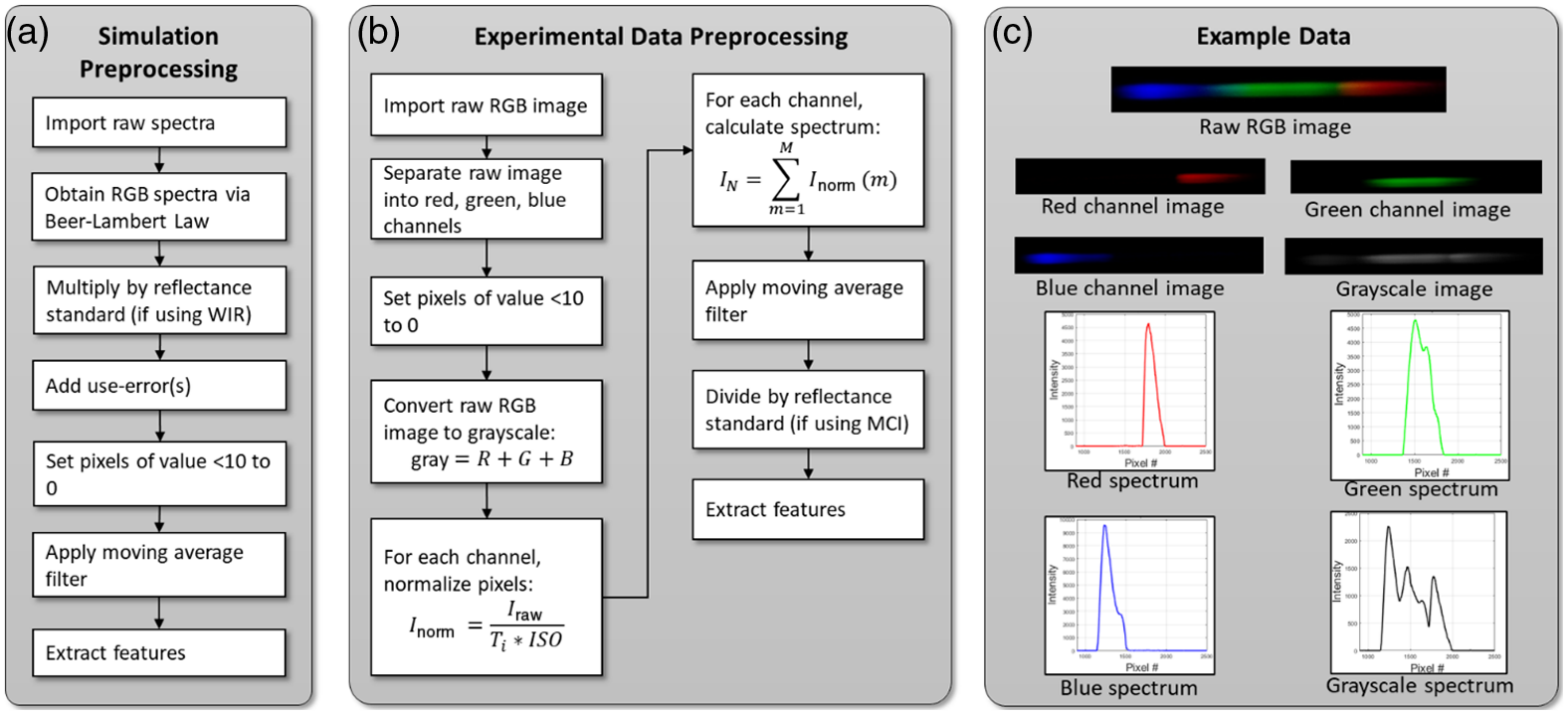
Preprocessing protocol for (a) simulated and (b) experimental data. The moving average filter for experimental data used a window size of 6. (c) Examples of the raw RGB images (for experimental data), as well as the red, green, and blue (RGB) spectra for both the experimental and simulated data, prior to normalization via reflectance standard. Variables are defined as follows. M: total number of pixel rows in an image and m: pixel row currently being evaluated. N: number of pixel columns (or wavelengths) in an image. R: red channel of 8-bit RGB image, G: green channel, and B: blue channel of 8-bit RGB image. Gray: array that sums, element-by-element the values of the arrays R, G, and B. $I_{\text{norm}}$: normalized pixel value of each array R,G,B, and gray. $I_{\text{raw}}$: raw pixel value from the R, G, B, and gray arrays. $I_{N}$: spectral intensity at pixel column N.

The presented WIR model uses four spectra to extract features [Fig. 3(c)]. (1) The spectrum from the (1) red channel, referred to as the “red spectrum;” (2) green channel or the “green spectrum;” (3) blue channel or the “blue spectrum” of the image; and (4) the spectrum from the grayscale image (the “grayscale spectrum”). Because the output of the forward Monte Carlo model is what the WIR model sees as the grayscale spectrum, an additional step was necessary during simulation preprocessing [Fig. 3(a)], that included converting the “grayscale” simulated spectra to the red, green, and blue (RGB) spectra. This was done by utilizing a modified version of a Beer–Lambert based, wavelength-to-RGB calculator, available by the Academo Organization.^30^ Specifically, the predicted RGB intensity at each wavelength was converted to a percent. For example, at the wavelength of 500 nm, the RGB coordinates are (0, 255, 146). These were converted to percentages of the overall signal strength attributed to each channel, or (0, 0.64, 0.36). The spectral intensity of the “grayscale” spectrum at 500 nm was then divided into three channels, with intensities proportional to these RGB percentages at each wavelength. Features were extracted from these red, green, and blue spectra for input into the WIR model.

Once both the simulated DRS “image” and its spectrum had been obtained, if the data was to be input into the WIR model, the spectrum was multiplied, column-by-column, by the spectrum of a reflectance standard. This was done to account for the general impact of the light source itself on the spectral values. By including this into the training set of the simulated dataset, the need for users to calibrate the system with a reflectance puck in the clinic was minimized. Feature extraction took place from both the RGB images, and the spectral graphs of the phantoms, according to Table 2. To validate the WIR model’s performance on these simulated datasets, a fivefold cross-validation technique was used, where the training and testing sets consisted of randomly selected samples from the corresponding simulated dataset. For example, to evaluate how well the algorithm performed on perfect + Gaussian noise data, randomly selected samples from the 1520 sample-dataset of the perfect + Gaussian noise dataset were used for training/testing.

**Table 2:** Features extracted and employed to predict optical properties from DRS data.

Feature Family	Feature
Spectral count values (12 features)	Maximum R, G, B, and grayscale spectral values
	Mean R, G, B, and grayscale spectral values
	Standard deviation of R, G, B, and grayscale spectral values
Spectral slope values (16 features)	Maximum R, G, B, and grayscale spectral slopes
	Minimum R, G, B, and grayscale spectral slopes
	Mean R, G, B, and grayscale spectral slopes
	Standard deviation of R, G, B, and grayscale spectral slopes
Other spectral values (6 features)	Spectrum length No. 1: number of pixel columns with intensity >0.25*maximum intensity value of RGB, grayscale spectra
	Spectrum length No. 2: distance (in pixels) between absolute maximum and second-highest maximum of grayscale spectrum
RGB channel values(12 features)	Skewness of R, G, B, and grayscale spectra
System configuration (one feature)	Probe geometry (categorical, probe 1 or 2)

### 2.3 Experimental Data Collection

One important consideration to ensure that a supervised model will be clinically translatable is having a realistic training dataset. To accomplish this, 882 DRS images were captured from 170 tissue-mimicking phantoms. This data were collected from 11 different experiments using the SmartME system. The exact number of images that were captured at each titration varied, but generally included taking at least three images at each titration. If multiple camera settings (i.e., exposure times and/or ISO speeds) were used within one experiment, generally three images were captured at each camera setting, for each phantom.

Phantoms included a range of hemoglobin concentrations ranging from 2.57 to 32.7  μm, resulting in theoretical $\mu_{a}$ ranging from 0.17 to $3.02\;{\mathrm{cm}}^{-1}$ and theoretical $\mu_{s}^{\prime }$ ranging from 4.98 to $14.69\;{\mathrm{cm}}^{-1}$, to mimic that of normal to cancerous cervical tissue.^6^ Phantoms contained deionized water, polystyrene spheres (Polysciences, Cat No. 0719-15, diameter=1.0  μm), and human hemoglobin powder (Sigma Aldrich, SKU H0267, CAS No. 54651-57-9). The protocol for data collection detailed by Hong et al was followed for data acquisition, using the SmartME system for data acquisition.^5^ Eleven of these experiments were conducted, over a span of 2 years.

To extract the theoretical optical properties of each titration, a sample from the stock solution was measured with a commercial-grade spectrophotometer at the beginning of each experiment (either Perkin-Elmer Lambda 35 or Thermo-Fisher SPECTRONIC 200). The expected optical properties for each consequent phantom were calculated by scaling the measured optical properties to the dilution value of the phantom solution. The theoretical value of $\mu_{s}^{\prime }$ for each phantom was calculated using Mie theory of light scattering.^31^

### 2.4 Phantom Data Preprocessing and Feature Extraction

The raw data collected by the SmartME DRS channel were an 8-bit RGB image [Fig. 1(a)]. To predict phantom optical properties, the preprocessing algorithm summarized in Fig. 3(b) was used. Specifically, once the data were collected, background noise was removed by passing the spectra through a thresholding algorithm, where all RGB pixels with an empirical intensity of <10 for a given channel in the raw image were set to 0 for that channel. For example, if an input pixel had RGB values of (0 20 9), the threshold algorithm would return (0 20 0) for that pixel. All images [Figs. 3(b) and 3(c)] were normalized on a pixel-by-pixel basis to account for differences in camera settings using Eq. (1), where $I_{\text{norm}}$ was the normalized pixel intensity, $I_{\text{raw}}$ was the raw pixel intensity, $T_{i}$ was the exposure time in seconds, and ISO is the ISO speed

$$I_{\text{norm}}=\frac{I_{\text{raw}}}{T_{i}\cdot \mathrm{ISO}}.$$ (1)

After this, the spectra were calculated using Eq. (2), where $I_{N}$ was the intensity at a given pixel column number and M was the number of pixel rows. Then, a moving average filter with a window size of six pixels was applied to the spectrum, for further noise reduction

$$I_{N}=\overset{M}{\underset{m=1}{\sum }}I_{\text{norm}}(m).$$ (2)

**Fig. 4:**
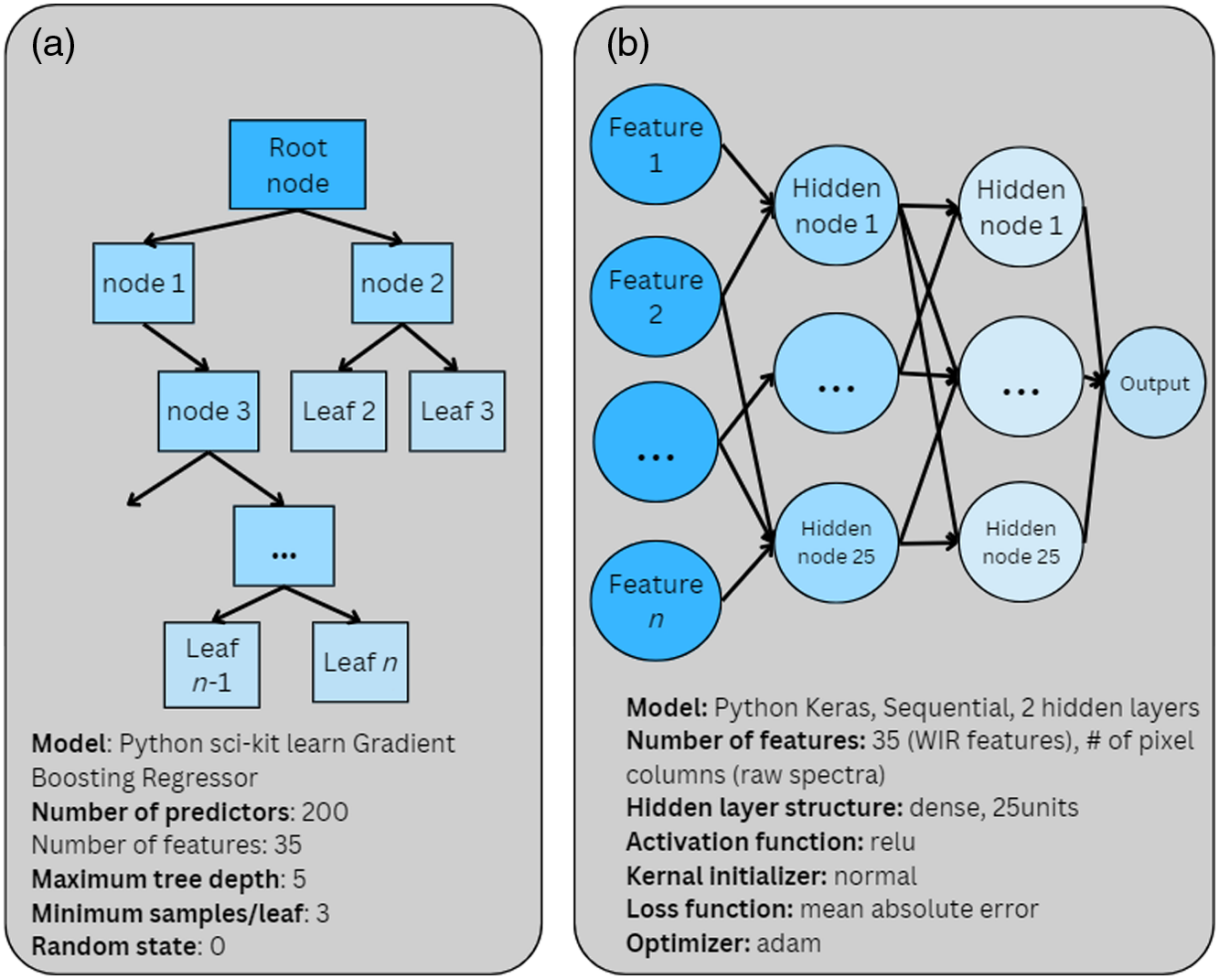
Architecture of (a) the WIR model and (b) the sequential dense NN that was used to analyze the DRS data. The MLP and fully sequential NN were used as baselines to simulate how models similar to those presented in previous literature may behave under the presented datasets.

Once data preprocessing was complete, feature extraction was performed per Table 2. The resulting feature table then served as input to a gradient boosting regressor from Python’s “sklearn.ensemble” library.^32^ This regressor is a tree-based model, with its structure illustrated in Fig. 4(a). It employed 200 estimators, at a max tree depth of 5, and a minimum of 3 samples per leaf. For repeatability, the random state was set to 0. Python version 3.9.1 was employed for all data preprocessing and model development. Data preprocessing, feature extraction, and optical property prediction were completed on an Intel(R) Core(TM) i5-7300U CPU @ 2.60 GHz 2.71 GHz processor, with 16.0 GB of RAM, running Windows 10.

To provide a baseline to how models like previous literature may perform on the proposed dataset, dense NN with 2 hidden layers was also employed using Python’s Keras library, with 25 nodes/layer [Fig. 4(b)].^33^ The average prediction of these models was used for calculating the prediction accuracy. Training of these models were done twice. First, the WIR features were used as input into the model and then the raw one-dimensional RGB DRS spectra were used as features. This included concatenating the grayscale spectrum, red channel spectrum, green channel spectrum, and blue channel spectrum to form one vector per sample. This was done as previous literature uses the raw DRS spectra as input to their models, and analyzing the DRS data in this fashion allows for one to fully compare the performance of the WIR model to models in literature.

### 2.5 Regression Training and Validation for Experimental Data

To augment the training process of the WIR model, 21,820 simulated datapoints were used. These included the perfect data (1520 datapoints, Table 3, dataset 1), datasets containing one of the previously described spectral artifacts (namely noise, intensity fluctuations, or wavelength miscalibrations) introduced (7600 datapoints, Table 3, datasets 2 to 5) and the dataset with all errors compounded on each other (1520 datapoints, dataset 6, Table 3). The remaining 11,180 simulated spectra that were used to augment WIR training for experimental data analysis included various combinations of errors (Table 3, simulated datasets 7 to 14).

**Table 3:** Simulated datasets used to enhance the training of the WIR model for analysis of experimental data. In some cases, multiple errors were compounded onto each other. Each simulated dataset contained 1520 simulated spectra. In total, 21,280 simulated datapoints were used to enhance training.

Simulated dataset No.	Gaussian noise (SNR = 35:1)	Scaling (spectra systematically scaled up by random amount, up to 5%)	Shifted (wavelength axis shifted left b 4.8 nm)	Compressed (spectrum moved inwards by 1 nm at tails)	Rotated (spectrum rotated by random amount, up to 5% at tails)
1					
2	×				
3			×		
4		×			
5				×	
6					×
7	×			×	
8	×		×		
9	×	×	×		
10	×	×	×	×	
11	×	×	×	×	×
12		×		×	
13		×	×		
14			×	×	

To evaluate the wavelength-independent WIR model’s performance on experimental data, either a leave-one-titration-out, or a leave-one-experiment-out approach was used. In the leave-one-titration-out approach, all data for a specified phantom titration was allocated to the testing set, and the simulation data outlined in Table 3 along with all other experimental data was assigned to the training set. This process was repeated for each of the 170 independent phantoms. For the leave-one-experiment-out approach, all data collected on 1 day was allocated to the testing set, and all other data (simulated and experimental) was used for training. This was repeated for each of the 11 experiment dates.

## 3 Results

Table 4 depicts the WIR model, the NN, and the MCI model’s performance at predicting $\mu_{a}$ and $\mu_{s}^{\prime }$, when various use-errors were introduced into the simulated dataset. Results for both models are presented as the mean root-mean square error (RMSE) of the prediction. The amount of time required to predict a test set of 304 samples with each model is also provided.

**Table 4:** Prediction accuracies of the MCI, WIR, and dense NN regressor models for optical property prediction of 1520 simulated spectra, with various use-errors. Results for the WIR model and dense NN were generated via fivefold cross-validation. Prediction times are provided for a testing set size of 304 samples. Bold text is used to indicate the lowest error value in each column.

Model	Prediction time (s)	Target	Perfect	Noise	Shifted	Intensity scaled	Com-pressed	Rotated	All
MCI (RMSE %)	n/a	$\mu_{a}$	**<0.01**	2.59	6.35	0.30	1.12	5.97	50.9
		$\mu_{s}^{\prime }$	**<0.01**	1.10	1.99	1.56	0.55	2.91	26.7
WIR (RMSE %)	$8.14\times 10^{-4}$	$\mu_{a}$	0.13	0.79	**0.26**	**0.16**	**0.19**	1.09	1.75
		$\mu_{s}^{\prime }$	0.27	0.80	**0.18**	1.18	0.12	0.70	1.53
		$\mu_{s}^{\prime }$	2.51	2.66	2.46	2.41	2.12	3.07	1.66
Dense NN - WIR features	0.146	$\mu_{a}$	0.86	2.18	1.05	1.98	1.61	10.9	2.24
		$\mu_{s}^{\prime }$	2.68	3.95	3.23	3.45	3.23	2.77	5.82
Dense NN - Raw spectra	0.199	$\mu_{a}$	0.17	**0.29**	0.84	0.95	**0.19**	**0.17**	**0.39**
		$\mu_{s^{\prime }}$	0.02	**0.07**	0.27	**0.08**	**0.1**	**0.56**	**0.12**

When a single error was introduced to the dataset, the MCI model was especially sensitive to shifting, which resulted in a 6.35% error for $\mu_{a}$ and rotating, which produced 5.97% error for $\mu_{a}$. However, the MCI model was relatively immune to noise, intensity scaling, and spectral compression. When all errors were compound, the MCI model reported RMSE of 50.9% and 26.7% for $\mu_{a}$ and $\mu_{s}^{\prime }$, respectively. Conversely, all machine learning models were more robust to various use-errors than the MCI model. For example, both the WIR and dense NN predicted optical properties with enough accuracy to be clinically useful (<10% RMSE for both $\mu_{a}$ and $\mu_{s}^{\prime }$) even when all use errors were compounded. The WIR model was arguably the most stable model with respect to use-error. This is seen as the prediction error was <2% for all use-errors. The dense NN when predicting $\mu_{a}$ for both the WIR features, and raw spectra as input had a prediction accuracy that varied by over 10% between the dataset with the lowest errors and the dataset on which the model performed the best, versus the worst.

Beyond the error-by-error variability seen in each’s model’s performance, the WIR model was more accurate than the dense NN when using the hand-picked features for all simulated datasets. While the dense NN, when using the raw DRS spectra as input yielded lower errors than the WIR model, this came at the expense of prediction time. Namely, the WIR model was able to render predictions in less than a millisecond for a testing set of 304 samples. The dense NN, on the other hand, was over 240 times slower, requiring 0.199 s to perform the same calculation. As a result, the WIR model is seen to provide the optimal balance between training time and model accuracy.

The most important features considered by the WIR model when predicting the optical properties of the simulated input spectrum, which included all of the errors compounded on each other, were also identified. The length of the grayscale spectrum, the mean slope of the grayscale spectrum, and the skewness of the green channel were the three most important features to predicting $\mu_{a}$. The three most important features to predict $\mu_{s}^{\prime }$ were the mean and standard deviations in intensity of the spectrum, as seen by the green channel, as well as the mean intensity of the grayscale spectrum.

Table 5 demonstrates the WIR model’s performance (the model that provided the optimal balance between accuracy and training time on the simulated data), compared to the MCI model (the current “gold standard” for optical property prediction of DRS data), on the experimental dataset. When using the leave-one-experiment-out validation method, the average percent RMSE for the WIR model was 17.8% and 19.3% for $\mu_{a}$ and $\mu_{s}^{\prime }$, respectively. For the leave-one-titration-out validation, the mean percent RMSE for the WIR model was 13.2% and 6.77% for $\mu_{a}$ and $\mu_{s}^{\prime }$, respectively. The MCI model produced prediction errors of 105% and 48.3% for $\mu_{a}$ and $\mu_{s}^{\prime }$, respectively, on the same dataset.

**Table 5:** Accuracy of wavelength-independent regression model at predicting optical properties of tissue-mimicking phantoms, where LOTO represents leave-one-titration out and LOEO represents leave-one-experiment out.

Date No.	Probe No.	Box No.	Mean % RMSE $\mu_{a}$			Mean % RMSE $\mu_{s}^{\prime }$		
			WIR LOTO	WIR LOEO	MCI	WIR LOTO	WIR LOEO	MCI
1	1	2	15.8	21.5	99.1^a^	7.80	13.5	18.4^a^
2	1	2	11.9	24.8	40.6^a^	8.48	27.1	11.5^a^
3	1	2	11.5	18.5	162^a^	4.21	15.4	74.2^a^
4	1	1	9.24	4.00	34.6	11.2	25.7	16.4
5	1	1	12.1	33.5	99.9	7.00	47.7	33.2
6	1	1	18.2	15.5	225	10.9	13.1	173
7	1	1	26.1	14.4	262	7.40	6.00	53.1
8	2	2	12.5	17.6	51.6^a^	3.82	22.3	29.0^a^
9	2	2	7.62	1.63	96.0	3.82	8.08	68.0
10	2	2	9.86	15.2	58.0^a^	4.30	17.5	50.6^a^
11	2	2	8.5	29.2	21.7	5.54	15.8	4.16
**Mean**	**—**	**—**	**13.2**	**17.8**	**105**	**6.77**	**19.3**	**48.3**

a Results were generated by dividing spectra by the reflectance standard from the 9/17/2021 experiment, due to unavailability of a reflectance standard measurement from this date.

## 4 Discussion

Many studies have been conducted that evaluate the capabilities of machine learning to predict optical properties from DRS data.^11^^–^^24^ There is a gap, however, in validating these machine learning algorithms are able to produce clinically useful results on realistic datasets that inherently include use-error. The presented research addresses this gap by including simulations that capture the spectral artifacts, such as noise, wavelength miscalibrations, and intensity fluctuations, that come with common use-errors. Further, the presented research includes the largest, most diverse experimental DRS dataset, of which we are aware, that has been used to train and validate a predictive model for DRS optical property prediction.

### 4.1 Model Performances on Simulated Data

As noted in Table 4, the MCI model, under perfect conditions, provided essentially perfect optical property predictions (as expected, since this data were the untouched output of the forward model). However, the WIR algorithm outperformed both the MCI model and the NN models, in terms of accuracy and training time, when various use-errors were introduced into the simulated dataset.

The reason for the large difference between the performances of models can be understood by examining the methodology each algorithm uses to render its predictions. The MCI model, for example, makes its predictions by comparing, wavelength-by-wavelength, the intensity of the measured spectrum, compared to what is theoretically expected for a given wavelength, at a given absorption and scattering level. Once there is convergence between the measured and spectra, the optical properties of the converged theoretical spectrum are returned. Since this method is deeply rooted in the theory of photon transport in turbid media, when the data input into the inverse model perfectly complies with theory, the model is essentially errorless, as seen in the leftmost column of Table 4, when perfect data were entered. However, experimental data rarely, if ever, perfectly complies with theory. Noisy data due from sources, such as camera noise, is inevitable. Wavelength shift due to optical misalignments between calibrations is realistic. Spectral intensity changes due to a failure to properly warm up, charge, or keep cool, a light source can occur, even to the most diligent technicians on the most well-maintained systems. Because the MCI model and similar models do not include these use-errors when analyzing spectroscopy data, when these errors, either individually or in combination, are present in data, the MCI model and similar algorithms produce large prediction errors, such as those seen in Table 4.

Machine learning models, like the ones presented here and similar models described in previous literature,^11^^–^^24^ provide an improvement upon the MCI model for optical property prediction. This is likely because machine learning models are naïve to the appearance of theoretical spectra. In the case of machine learning, these models simply evaluate the input data, and make correlations between changes in spectral features and changes in the assigned optical properties. Due to this, if error (especially a systematic one) is present in the data acquisition process, this is not necessarily seen as an error or an artifact by the model, and as a result, this provides a level of robustness to error among machine learning models.

The major shortcoming of using NNs is that the models, because they must both extract features and assign a target variable, take a long time to complete the training process. Additionally, the computational complexity of NN models are greater than a traditional machine learning approach, such as the WIR model. Due to this, NNs require more training data than traditional machine learning models to avoid overfitting. The end result of this is that the MLP models tend to be more sensitive to deviations in the appearance of testing data from their training set, as demonstrated by the NNs being more prone to variable prediction accuracies, depending on the use-error introduced to the dataset.

The presented WIR algorithm, on the other hand, on top of embracing and anticipating use-error in analyzing spectroscopy data, can yield lower errors after being trained on a simpler dataset (i.e., 35 hand-selected features, instead of the raw RGB signals), and produce optical property predictions after training in less time than NNs. This is accomplished in two major ways, (1) the method determines which features are extracted from the measured data, and (2) the WIR algorithm defines what “theoretical” data looks like. First, the WIR algorithm, as mentioned in Table 2, examines the spectrum holistically, rather than on a wavelength-by-wavelength basis. The most important features the WIR model used to render a prediction for $\mu_{a}$ were the length (in pixel columns) of the spectrum, the mean slope of the grayscale spectrum, and the skewness of the green spectrum. This is likely because the overall signal detected by the DRS system will be weaker with increased $\mu_{a}$. With this, the local minima and maxima of the spectrum will be less pronounced and will manifest as a smaller mean slope of the spectrum. Further, because the detected signal will be weaker with increased $\mu_{a}$, the difference between the maximum spectral intensity, and 25% of the spectral intensity (the definition of the most important feature for $\mu_{a}$ prediction), will be smaller. While these features will change as the optical properties of the tissue change, none of these feature values will change significantly if the wavelength axis is misaligned, the spectrum is noisy, or the spectrum is systematically scaled up or down, explaining the model’s robustness to these use-errors. Errors, such as compressing the spectrum, which simulate misalignment among the spectrometer camera, the gratings, collimator, and the fiber optical connector, will impact the shape of the spectrum, but will not significantly impact the overall intensities of the spectrum, since these features, while not the top three most important features, are still used by the WIR model, and this can explain why the model was robust to these artifacts as well (Table 3).

With respect to $\mu_{s}^{\prime }$ prediction, the WIR model gave most weight to the mean and standard deviation in the intensity of the green spectrum, and the mean intensity of the grayscale spectrum. These, again, are values that will be less likely to change due to miscalibration artifacts, or noise. Further, alignment error manifesting as compression of the spectra will not seriously change the values of these features, either. As a result, the WIR model was robust to these errors.

Because there are enough features extracted that will not be severely impacted by any singular use-error in the WIR algorithm, the algorithm is robust to various errors, even when compounded on each other, as demonstrated in Table 3. By making a specific effort to extract features that will be consistent, despite the presence of these use-errors, the WIR algorithm is more robust than the MCI model.

### 4.2 Model Performances on Experimental Data

It can further be concluded that the presented algorithm is likely to be useful for predicting optical properties from experimental data, especially when validated with the previously described leave-one-titration-out validation approach. Under this approach, across 882 experimental datapoints, the WIR algorithm yielded prediction errors of 13.2% for $\mu_{a}$ and 6.77% for $\mu_{s}^{\prime }$. The MCI model produced errors an order of magnitude higher, of 105% for $\mu_{a}$ and 48.3% for $\mu_{s}^{\prime }$. It is anticipated the reason behind this large difference in each algorithm’s performance is similar to that seen in the simulated data. All experimental data, including the dataset presented here, includes random noise. Sometimes, a measurement from calibration standard was not available on the same day of experimentation, which may have contributed to wavelength-dependent errors in evaluating the spectral intensity. While a wavelength calibration was performed for each experiment, the wavelength calibration was not necessarily perfect. Because the WIR model incorporates these artifacts into its training process, and because the WIR model uses features that are inherently more robust to these use-errors than the MCI model, the WIR model was able to outperform the MCI model on this experimental dataset.

A limitation of these results includes that the errors of the phantom data are higher than that was seen using simulated data for both the WIR and MCI models. This can be attributed to the hardware and methodology used to collect this data. Specifically, the experimental data were collected on an in-house built SmartME system.^5^ This device was an experimental device that was continually being destructed and reconstructed throughout the 2-year span in which the experimental data were collected. As a result, a limitation in the experimental dataset is that there were some large day-by-day dependences regarding the characteristics of the experimental spectra, explaining why the algorithm saw higher errors in predicting the optical properties using the leave-one-experiment-out method, and why the MCI model saw large errors overall. Even within a single day, the experimental data were collected on an experimental, not commercialized, system, and sometimes data were collected incorrectly (i.e., data were collected on a light source that was not properly warmed up or charged). There were errors introduced into the dataset that are related to data collection on an incomplete device, rather than the typical use-errors that are outlined in this paper.

Nevertheless, the data were included in this manuscript for four reasons: (1) to demonstrate how sensitive the MCI model is to common use-errors; (2) to demonstrate how the WIR model is able to be trained faster, and be more robust to various use-errors than a NN; and (3) to present the first study, to the best of knowledge, to include data collected on multiple days for machine learning model training of optical property prediction from DRS data. Because this, the first time data like this have been presented, and it is entirely possible that the algorithms presented by other groups may be vulnerable to similar day-by-day dependencies in their datasets, however, these groups did not include enough experimental data to detect this trend. Thus, it is important to quantify and draw attention to this potential limitation of machine learning models for optical property prediction, such that this can inform experimental design for research groups in the future. Future work will be focused on collecting another extensive, experimental dataset that provides more day-by-day consistency in the quality of the DRS data, to further validate the previously described algorithm. That said, due to the robustness of WIR model to the experimental data presented thus far, if the algorithm can yield reliable results under these inconsistent experimental conditions, it is likely to be clinically translatable on clinical/experimental data that are collected on commercial-grade systems.

## 5 Conclusion

The presented WIR algorithm presents reliable optical property predictions from DRS data. This algorithm is robust to various artifacts that come from common use-errors, such as noise, intensity fluctuations, wavelength miscalibrations sensor, calibration, and light source errors. This algorithm has also been rigorously validated on an extensive experimental dataset, showing promise for its translatability to a clinical setting.

## Data Availability

The experimental data and code that support the findings of this article are not publicly available due to a pending patent application made on behalf of the experimental SmartME device. They can be requested from the author at bing.yu@marquette.edu.
