# Designing a use-error robust machine learning model for quantitative analysis of diffuse reflectance spectra (Erratum)

**DOI:** 10.1117/1.JBO.29.11.119801

**Published:** 2024-11-27

**Authors:** Allison Scarbrough, Keke Chen, Bing Yu

**Affiliations:** a Marquette University, Medical College of Wisconsin, Joint Biomedical Engineering Department, Milwaukee, Wisconsin, United States; b Marquette University, Computer Science Department, Milwaukee, Wisconsin, United States

## Abstract

The erratum documents a correction to the originally published article. The original article was corrected and republished 18 November 2024, doi: https://doi.org/10.1117/1.JBO.29.1.015001.

This article [*J. Biomed. Opt.* **29**(1), 015001 (2024) doi: 10.1117/1.JBO.29.1.015001] was originally published on 11 January 2024 with an error in the calculation of the RSME. The article was corrected and republished 18 November 2024.

In the original version, mean errors for the wavelength-independent regressor (WIR) model were reported as 13.2% and 6.1% for $\mu_{a}$ and $\mu_{s}^{\prime }$, respectively. In the corrected version, those values are 12.5% and 5.77% for $\mu_{a}$ and $\mu_{s}^{\prime }$, respectively.

In Table 5, the average RMSE percentage for the leave-one-experiment-out (LOEO) validation of the WIR model is 24.2% and 13.2% for $\mu_{a}$ and $\mu_{s}^{\prime }$, respectively, rather than the originally reported 17.8% and 19.3%. For the leave-one-titration-out (LOTO) validation method, the mean percent RMSE for the WIR model is 12.5% and 5.77% for $\mu_{a}$ and $\mu_{s}^{\prime }$, respectively, rather than the originally reported 13.2% and 6.77%.

